# Supplementary material for: The interactome of intact mitochondria by cross-linking mass spectrometry provides evidence for coexisting respiratory supercomplexes
Source: Mol Cell Proteomics. 2017 Dec 8;17(2):216–32. doi: 10.1074/mcp.RA117.000470 (PMC5795388; doi:10.1074/mcp.RA117.000470)
Supplement: Supplemental Data [file supp_RA117.000470_133922_0_supp_23978_fzffwf.zip › spectra_annotation/mito_DR_spectra_annotation/433-1-5-1-10-1.pdf]

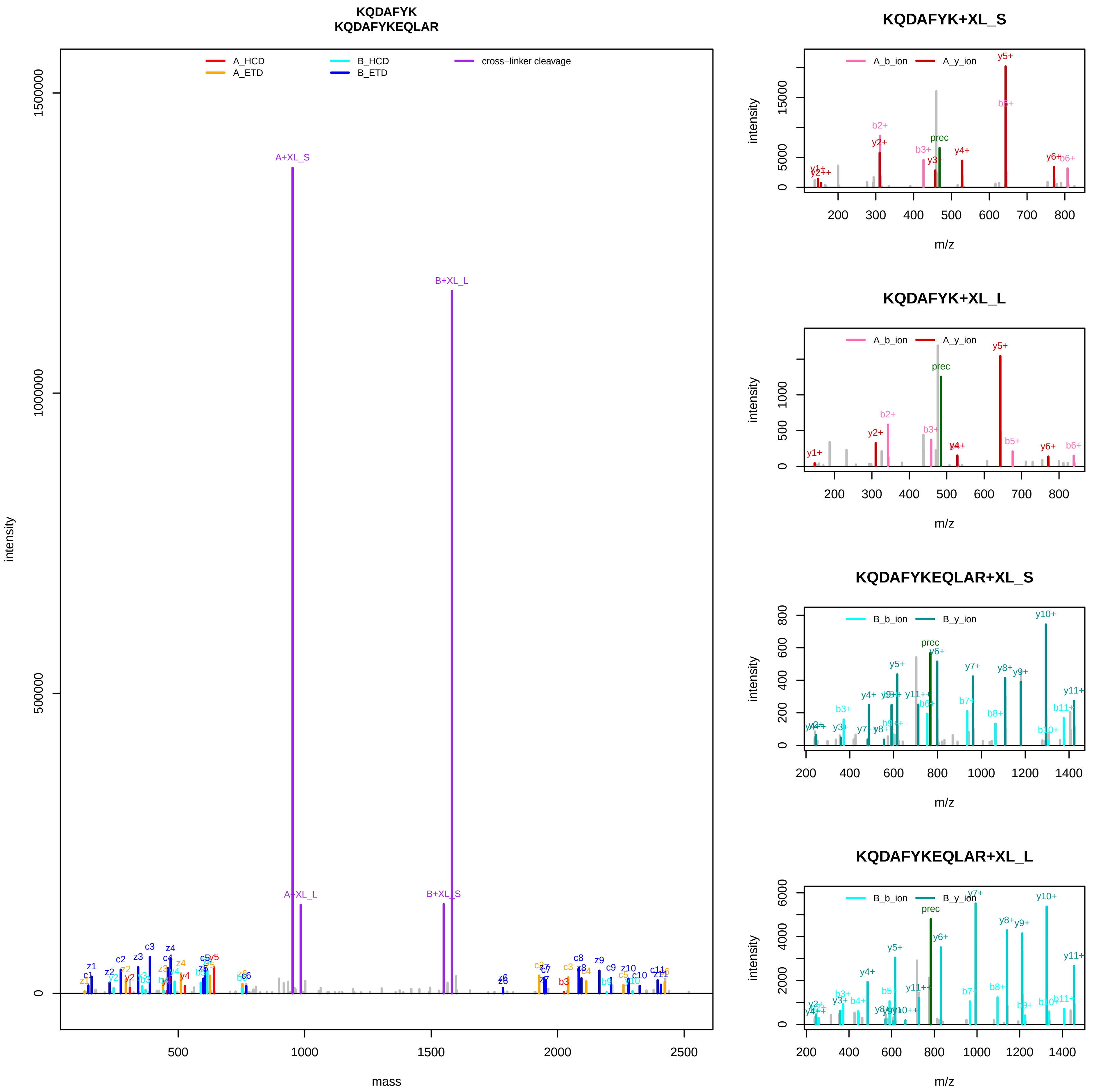
KQDAFYK
KQDAFYKEQLAR
A_HCD
A_ETD
B_HCD
B_ETD
cross−linker cleavage
A+XL_S
B+XL_L
A+XL_L
B+XL_S
intensity
mass
0
500000
1000000
1500000
500
1000
1500
2000
2500
KQDAFYK+XL_S
KQDAFYK+XL_L
KQDAFYKEQLAR+XL_S
KQDAFYKEQLAR+XL_L
A_b_ion
A_y_ion
B_b_ion
B_y_ion
prec
m/z
